# Detections of rare enterovirus C105 linked to an emerging novel clade, Spain, 2019 to 2024

**DOI:** 10.2807/1560-7917.ES.2025.30.6.2500073

**Published:** 2025-02-13

**Authors:** Maria Dolores Fernandez-Garcia, Juan Camacho, Francisco Diez-Fuertes, Estrella Ruiz de Pedro, Nerea García-Ibañez, Ana Navascués, Carla Berengua, Pedro Antequera-Rodriguez, Montserrat Ruiz-García, Maria Teresa Pastor-Fajardo, María Cabrerizo

**Affiliations:** 1 Centro Nacional de Microbiología, Instituto de Salud Carlos III, Madrid, Spain; 2 Centro de Investigación Biomédica en Red (CIBER) Epidemiology and Public Health (CIBERESP), Madrid, Spain; 3 Centro de Investigación Biomédica en Red (CIBER) Infectious Diseases (CIBERINFEC), Madrid, Spain; 4 Hospital Universitario de Navarra, Pamplona, Navarra, Spain; 5 Hospital Universitario de la Santa Creu i Sant Pau, Barcelona, Spain; 6 Hospital General Universitario Morales Meseguer, Murcia, Spain; 7 Hospital General Universitario de Elche, Alicante, Spain

**Keywords:** Enterovirus C, molecular surveillance, acute flaccid paralysis

## Abstract

Enterovirus (EV)-C105 is a rare genotype not previously detected in Spain. Between 2019 and 2024, we detected EV-C105 in respiratory samples of five patients, through routine EV surveillance. Three cases had respiratory illness and two were hospitalised for neurological illness. Four of the five sequenced strains belonged to an emerging clade (C1), defined by four novel nonsynonymous mutations in key antigenic epitopes. We recommend reinforced clinical awareness and EV genomic surveillance, including respiratory samples, even when symptoms are neurological.

Enterovirus C105 (EV-C105) is a rare genotype within enterovirus species C. It was first identified in 2010 in the Democratic Republic of the Congo (DRC) from a patient with fatal acute flaccid paralysis (AFP) [1]. Subsequently, between 2010 and 2018, sporadic EV-C105 strains were detected worldwide, mostly associated with respiratory disease, although the overall prevalence appears to be low [2-6]. However, since 2023, the United Kingdom (UK) and some other European countries (Italy, Slovenia, the Netherlands and Belgium) observed an increased detection (European Non-polio Enterovirus Network (ENPEN) communication) [7]. Scarce EV-C105-associated clinical data are available. Here we describe the first detections of EV-C105 in Spain in patients with neurological and respiratory disease, along with the clinical and virological characteristics of the upsurge.

## Detection and molecular characterisation

Samples included in the analysis were collected through a voluntary non-polio enterovirus (NPEV) surveillance system coordinated at the National Reference Enterovirus Laboratory (EVL). Hospitals send EV-positive specimens to the EVL from patients with clinical manifestations associated with EVs (mainly neurological, respiratory, cardiac and skin-related symptoms), regardless of severity. Detection and genotyping methods at the EVL include reverse-transcription nested PCRs targeting the 5′-untranslated region (UTR) and VP1, respectively, as previously described [8-10], followed by Sanger sequencing and Basic Local Alignment Search Tool (BLAST) analysis.

Between January 2019 and December 2024, 2,443 EV-positive samples were sent to the EVL for genotyping (523 in 2019, 90 in 2020, 204 in 2021, 395 in 2022, 478 in 2023 and 753 in 2024). Enterovirus-C105 was identified in five (0.2%) EV-positive samples: one in 2019, two in 2023 and two in 2024.

All EV-C105 samples were detected in hospitals using different real-time multiplex PCR panels for respiratory viruses (BioFire FilmArray, bioMérieux, Marcy-l'Étoile, France; Vitro Master Diagnostica, Granada, Spain) or for EV-parechovirus (Progenie Molecular, Valencia, Spain). We obtained whole genome sequences directly from EV-C105-positive samples by using a metagenomics-based approach, as previously described [11]. In two of the EV-C105-positive samples, reads corresponding to other coinfecting viral pathogens (rhinovirus) were found by de novo assembly, as presented in Supplementary Table 1.

## Epidemiological and clinical features

All five EV-C105 strains were identified in throat swabs. Of note, the stool sample culture from a patient with AFP (EV1978), on RD cells, a human rhabdomyosarcoma-derived cell line commonly used for EV isolation, tested negative. Infections with EV-C105 were diagnosed in children aged 2–10 years. Three patients presented with respiratory symptoms. The other two patients presented with neurological conditions (meningitis and AFP), both requiring treatment in an intensive care unit for 5–30 days. The male:female ratio was 4:1. Cases originated from four different geographically distant provinces in Spain (Alicante, Barcelona, Murcia and Navarre).

## Phylogenetic analyses

**Figure 1:**
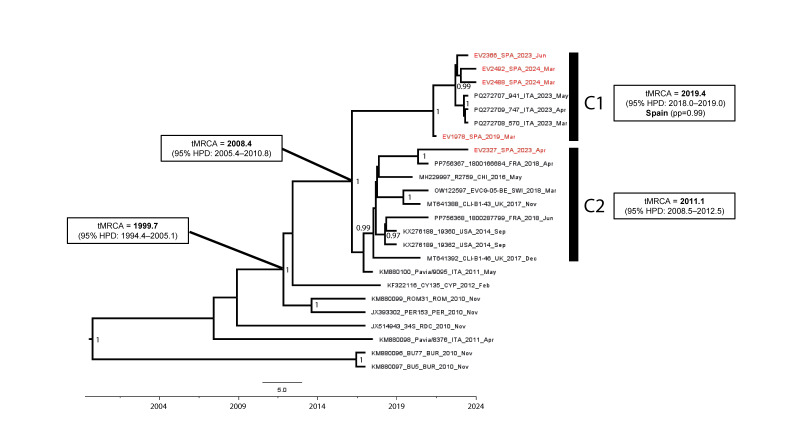
Bayesian-inferred phylogenetic tree with the most likely time of the most recent common ancestor for enterovirus C105 strains, Spain, 2019–2024 (n = 5)

HPD: highest posterior density; MRCA: most recent common ancestors.

Since recombination events can influence tree topologies, we first tested our EV-C105 global dataset for recombination using SimPlot, version 3.5.1 (https://sray.med.som.jhmi.edu/SCRoftware/SimPlot/). We did not detect recombination events with other EV-C strains that could have led to the EV-C105 upsurge. Then, to investigate the evolutionary history of EV-C105, maximum clade credibility trees were constructed based on complete VP1 sequences (Figure 1) and whole genome sequences generated in this study, along with a global EV-C105 sequence dataset. More details can be seen in Supplementary Figure 1. Sequences from four of the five strains clustered in a well-supported clade C1 (posterior probability (pp) = 1) with strains detected in 2023 in Italy showing high nt identities (> 99% and > 97% in VP1 and whole genome, respectively). According to Bayesian inference, the most recent common ancestor (MRCA) of C1 emerged, presumably in Spain (pp = 0.99), during 2019 (95% highest posterior density (HPD) interval: 2018–2019). This timeline aligns with the detection of the EV-C105 C1 strain (EV1978) in the patient with AFP. The other four EV-C105 C1 sequences were from patients with respiratory illness. The EV-C105 sequence EV2327 from 2023 grouped in C2 (95% HPD: 2008–2012) and was related to strains circulating in Europe, the United States (US) and China between 2014 and 2018. Both clades, C1 and C2, show a sequence divergence > 5% from each other, aligning with thresholds previously established for well-characterised EVs [12].

Maximum-clade credibility (MCC) tree of complete VP1 sequences included five strains of EV-C105 and sequences from all previously described EV-C105 strains extracted from GenBank (https://www.ncbi.nlm.nih.gov/genbank/) (dataset: 19 sequences available in GenBank on 8 Nov 2024). The analysis was performed with BEAST version 1.10.4 (https://beast.community) to estimate the date and location of the most recent common ancestors (MRCAs). The BEAST priors were introduced with BEAUTi version 1.10.4, including an uncorrelated relaxed molecular clock model with a lognormal rate distribution, the Bayesian skyline plot demographic model and the SRD06 nt substitution model. Markov chain Monte Carlo (MCMC) runs of 100 million states sampling every 10,000 steps were computed. The convergence of MCMC chains was checked using Tracer version 1.7.1 (https://github.com/beast-dev/tracer/releases), ensuring that the effective sample size (ESS) values were > 200 for each estimated parameter. The MCC tree was obtained from the tree posterior distribution using Tree-Annotator after a 10% burn-in. Similar phylogenetic MCC tree topology was obtained when the analysis was performed using whole genome sequences, as presented in Supplementary Figure 1. The best-fit evolutionary model was also estimated for VP1 sequences to reconstruct a maximum likelihood tree that also supports the sequence clustering obtained in the Bayesian analysis, as shown in Supplementary Figure 2. The EV-C105 strains from this study are indicated in red. Taxon information includes GenBank accession number, strain name, country of origin, year and month of detection.

## Amino acid sequence comparison

**Figure 2:**
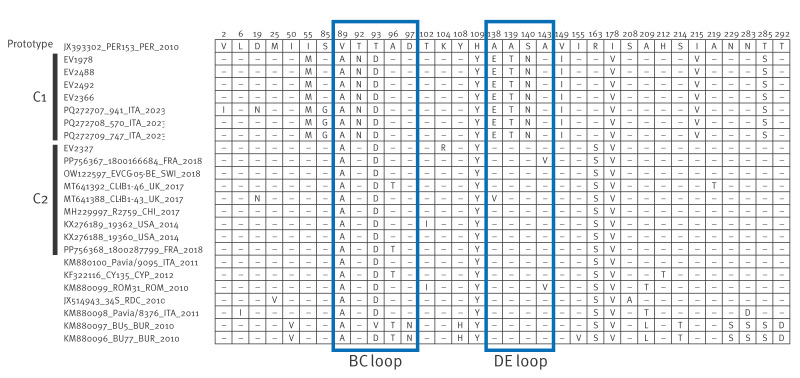
Alignment of deduced amino acid sequences (n = 296) of the VP1 polyprotein of enterovirus C105 strains, Spain, 2019–2024 (n = 5) with the prototype sequence (JX393302) and sequences of global strains extracted from GenBank (n = 19), 2010–2023, showing only variable sites

The deduced amino acid sequences of the VP1 region of all five EV-C105 strains were compared with prototype PER153 strain (JX393302). Unique nonsynonymous mutations in the DE loop (A138E, A139T, S140N) and the BC loop (T92N) of EV-C105 C1 strains were found when compared with global EV-C105 strains (Figure 2).

EV: enterovirus.

Dash represents amino acids identical to those in the prototype strain. Strains in red indicate study strains. Amino acids in the BC and DE loops are indicated in boxes as reference [3]. Unique amino acid mutations in C1 when compared with global EV-C105 strains are indicated in bold.

## Discussion

We report the detection of five EV-C105 strains between 2019 and 2024 from different geographic areas in Spain. This genotype had not previously been detected in Spain after the establishment of polio and non-polio enterovirus surveillance in the EVL with both cellular and molecular methods in 2006, suggesting its recent emergence in our country.

Most worldwide EV-C105 cases have been linked to respiratory infection [2,3,5,6]. However, two cases with AFP associated with this virus have been reported to date: one in the DRC in 2010 and another in the US in 2014, suggesting a neurotropic potential [1,4]. Consistent with this, we identified EV-C105 strains in three cases with respiratory illness and in two cases with neurological disease (hospitalised for meningitis and AFP), further supporting the virus potential to cause severe neurological disease. It is important to note that this association between EV-C105 and neurological disease may be limited by the detection of the virus only in respiratory samples. Nevertheless, the lack of detection in cerebrospinal fluid (CSF) does not exclude this possibility, as recognised neurotropic EVs, such as poliovirus, EV-A71 and EV-D68 (which, like EV-C105, are primarily associated with respiratory symptoms) are also rarely detected in CSF [13,14]. Further pathophysiological studies may be required to evaluate the neurotropic potential of EV-C105.

In our study, we also document the emergence of a novel clade, C1, which includes sequences from Spain and Italy predominantly 2023–2024. The high genetic similarity of EV-C105 C1 strains from these countries suggests rapid transmission. Changes in the amino acid composition of the main antigenic epitopes (BC and DE loops) [3,15] have been associated with clade divergence and with rapid transmission of EVs causing epidemics [16-18]. In line with this, we identified nonsynonymous amino acid substitutions in these loops of EV-C105 C1 strains, which may explain the rapid transmission observed 2023–2024. Still, the specific mechanism by which these mutations may have altered immune escape or virulence of EV-C105 requires further investigation.

Our phylogenetic analysis also reveals a replacement pattern where C2, predominant from 2014 to 2018, seems to have been displaced by the emerging C1, which has become dominant 2023–2024. This shift in dominance follows a typical pattern observed in other EV types, although earlier strains may persist in the population at lower rates [11,19,20]. In line with this, the detection of an EV-C105 C2 strain from 2023 suggests that the complete replacement of C2 by C1 has not occurred. This concurrent co-circulation of strains from different clades could have contributed to extensive antigenic diversity, potentially enabling EV-C105 strains to partially evade population immunity, thus explaining their observed emergence [18].

The parallel increase in cases in Europe since 2023 (ENPEN communication) [7] would suggest that the emergence of the C1 clade may have contributed to the spread of EV-C105 across Europe. To validate this, enhanced genomic surveillance is needed to expand the EV-C105 sequence database, allowing for a more comprehensive study of its molecular evolution. Furthermore, the case with AFP infected with EV-C105 C1, raises the necessity to reinforce clinical awareness of cases with neurological symptoms to assess if circulation of C1 strains has increased the occurrence of severe infections in children across Europe.

Careful consideration must be given to sample selection in neurological infections. Respiratory and stool specimens are considered the most appropriate sample types for EV diagnosis in patients with paralysis or myelitis [13]. Nevertheless, it is well-known that EV-D68, a respiratory EV associated with myelitis, is rarely detected in stool samples [5,21]. Similarly, the difficulty of detecting rare EV-Cs, such as EV-C105, EV-C116 and EV-C109 in stool samples has also been documented [1,4,5]. Our findings align with this evidence, as both of our cases with neurological illness were identified through respiratory samples, while the stool culture for the case with AFP (EV1978) tested negative. These results highlight the importance to promote collection of respiratory specimens, even when symptoms are neurological, to improve EV-C105 detection and potentially other neurotropic NPEVs.

The EV-C105 differs from other EV-C viruses by a divergent 5′-UTR, which poses challenges for detection, as most multiplex PCR panels that detect EVs target the 5’UTR [22]. However, in this study, three different panels and the 5’UTR nested-PCR used at the EVL [9] successfully detected EV-C105 strains, reassuring its detection despite the divergence. It remains unclear whether other multiplex PCR methods targeting 5’UTR would also detect EV-C105, potentially leading to false-negative results and underdiagnosis. Future studies on detection capabilities of the EV-C105 genome by commercial assays should be performed.

## Conclusion

In conclusion, the potential severity of EV-C105 infections causing neurological symptoms, combined with the emergence of a novel clade in Europe, underscores the need to strengthen clinical awareness and enhance EV surveillance in respiratory samples, even when the primary symptoms are neurological.

## Supplementary Data

513000 Supplementary Material
