# Supplementary material for: Detections of rare enterovirus C105 linked to an emerging novel clade, Spain, 2019 to 2024
Source: Euro Surveill. 2025 Feb 13;30(6):2500073. doi: 10.2807/1560-7917.ES.2025.30.6.2500073 (PMC11914964; doi:10.2807/1560-7917.ES.2025.30.6.2500073)
Supplement: Supplementary Material [file 25-00073_FERNANDEZ-GARCIA_Supplement.pdf]

Supplementary Material

This supplementary material is hosted by Eurosurveillance as supporting information alongside the article ‘Detections of rare enterovirus C105 linked to an emerging novel clade, Spain, 2019 to 2024’, on behalf of the authors, who remain responsible for the accuracy and appropriateness of the content. The same standards for ethics, copyright, attributions and permissions as for the article apply. Supplements are not edited by Eurosurveillance and the journal is not responsible for the maintenance of any links or email addresses provided therein.

Supplementary Figure 1. Bayesian time-scaled phylogenetic analysis with complete genome sequences of 5 enterovirus C105 study strains and sequences from all previously described enterovirus C105 strains extracted from GenBank with complete genomes (dataset: 15 complete sequences available at GenBank at 08 Nov 2024). The tree was performed with BEAST v1.10.4 to estimate the date and location of the most recent common ancestors (MRCAs). BEAST priors were introduced with BEAUTi v1.10.4, including an uncorrelated relaxed molecular clock model with a lognormal rate distribution, the Bayesian skyline plot demographic model and the SRD06 nucleotide substitution model. Markov chain Monte Carlo (MCMC) runs of 100 million states sampling every 10,000 steps were computed. Two independent runs were combined with LogCombiner v.1.10.4. and the convergence of MCMC chains was checked using Tracer v.1.7.1, ensuring that the effective sample size (ESS) values were greater than 100 for each estimated parameter. The maximum clade credibility (MCC) trees of the whole genome were obtained from the tree posterior distribution using Tree-Annotator after a 10% burn-in. Scale bar indicates nucleotide substitutions per site. EV-C105 strains from this study are indicated in red.

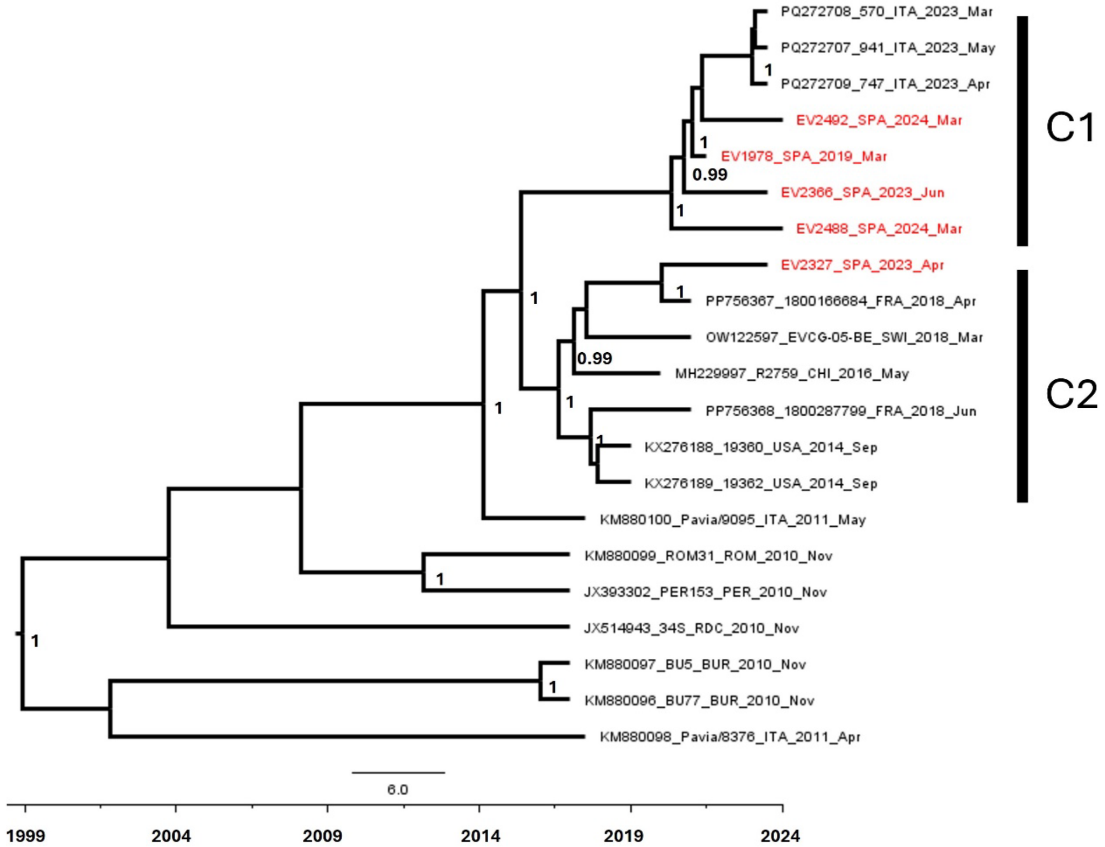

Supplementary Figure 2. The consensus tree of VP1 was constructed with the máximum likelihood method implemented in IQ-Tree web server using MZ092705 sequence as an outgroup. The best-fit evolutionary model selected by ModelFinder was TIM2e+I+G4 to VP1 and GTR+F+G4 to the whole genome according to the Bayesian Information Criterion. Only bootstrap values greater than 90 are showed.

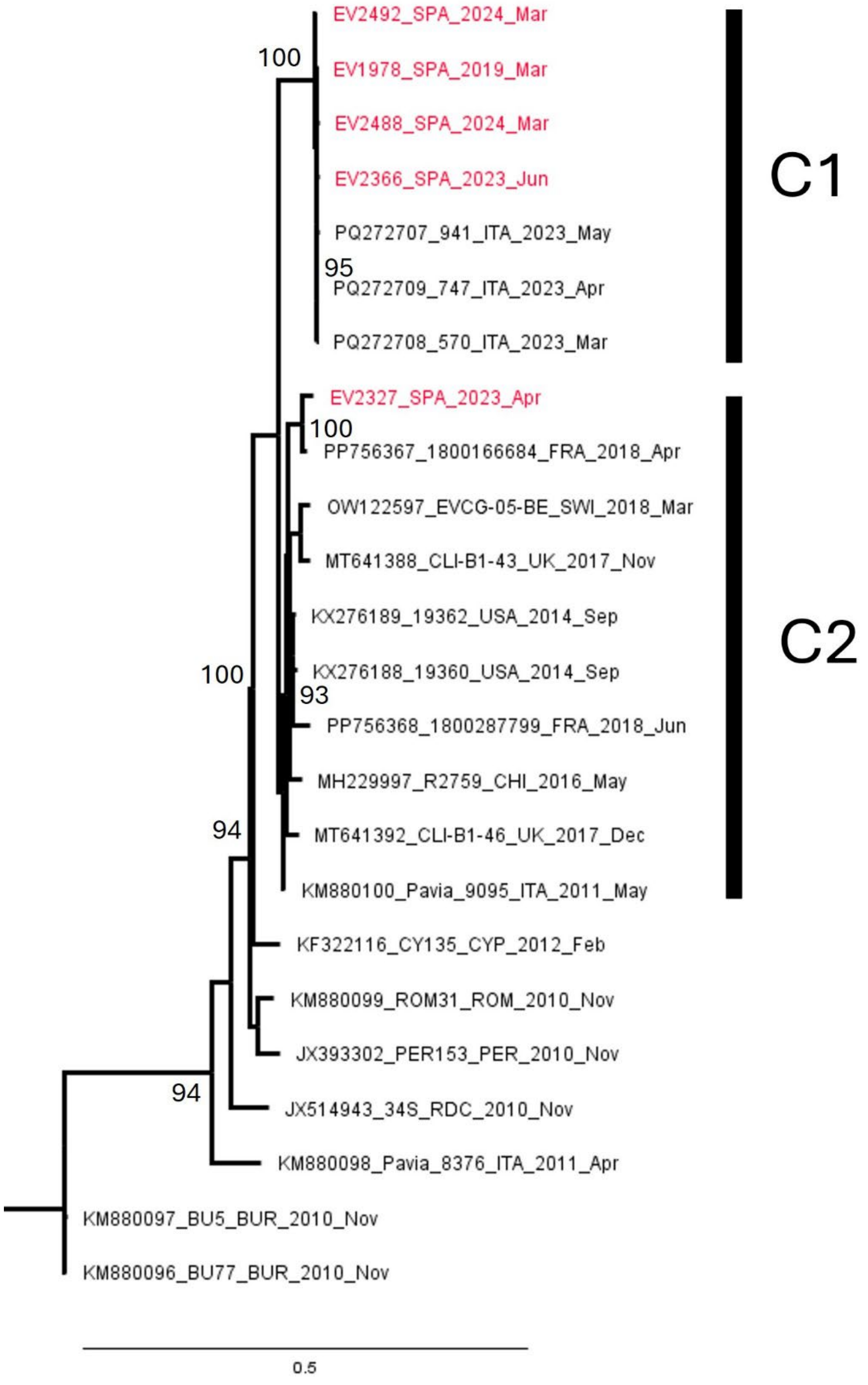

Supplementary Table 1. Results for metagenomic sequencing of enterovirus (EV) C105-positive samples from Spain

Strain designation	Virus detected	Accession No.	Total number reads	% viral reads	% host reads	Median coverage depth	Nt. length
EV1978	EV-C105	PV005818	4576904	24.48	74.92	20560	7347
	Rhinovirus A					39	698
EV2327	EV-C105	PV005821	2903842	0.76	90.93	62	7304
	Rhinovirus C					1634	7083
EV2366	EV-C105	PV005822	1688744	10.7	88.54	3400	7340
EV2488	EV-C105	PV005819	21644	14.88	43.2	56	7345
EV2492	EV-C105	PV005820	20472	25.61	70.4	95	7345
